# Pennsylvania State Society

**Published:** 1888-10

**Authors:** Ira G. Baumgardner


### Reports of Society Meetings.

### PENNSYLVANIA STATE SOCIETY.

### TWENTIETH ANNUAL MEETING.

REPORTED FOR THE INDEPENDENT PRACTITIONER.

BY IRA G. BAUMGARDNER, D. D. S.

CONTINUED FROM PAGE 493.

Discussion on paper read by Dr. E. C. Kirk, entitled "The Implantation of Human Teeth," and printed in September number.

*Dr. Gerhart*—I would like to ask Dr. Kirk how long those teeth had been extracted before they were implanted.

*Dr. Kirk*—I cannot give the ages of them. I have disregarded the question of the length of time the teeth had been extracted. They all had been out three months, and I may add that in the letter Dr. Herring wrote me, he stated that he had used a tooth he found in an old chest, the key of which had been lost for seventeen years. I do not think the age of the extracted tooth has any effect upon the operation.

*Dr. Gerhart*—I asked the question because I once used a tooth for transplantation that had been out for a long time. During this time of apparent death vitality seemingly remains in the periosteum. My friend, Dr. Beck, said the other day that he did not believe it possible that a tooth could be vital in a socket after extraction, and while I doubted it, yet I have had such experiences that it seems to me that it must have vital union. Even after the tooth had been extracted four months, and the question I would like to have answered is, whether in this apparently dead periosteum of the tooth vitality is capable of restoration at any period. If a tooth will remain alive for seventeen years, why not for 1700 years? And the question is whether it has the same vitality as the latent germ.

*Dr. Kirk*—I would like to ask Dr. Sudduth to answer this question, as he has worked up this subject very thoroughly.

*Dr. Sudduth*—See editorial in September number.

*Dr. Gerhart*—So far as Dr. Sudduth has answered my question, he has confirmed the opinion of Dr. Beck: it is a mechanical union. The reason I differed from Dr. Beck was on account of my own experience. Something like ten months ago, a young man presented himself with a central incisor which had been kicked loose by a horse. For several years it suppurated. He broke off the crown, and presented himself to have a crown put on; but on account of the great size of the apical foramen, I proposed transplantation of another tooth—one lying around for several years. Upon the extraction of the root, there was not a vestige of any alveolar process upon the labial aspect. The gum and the soft tissues were flaccid. The tooth I implanted was too small for the socket, and had to be held in place by means of ligatures. The tooth in position was a trifle longer than the mate in the mouth, and in the process of healing or attachment, or whatever you choose to call it, the tooth was drawn up—drawn up so as to be one-sixteenth inch shorter than the other teeth. I do not see how that could be accomplished except by vital action between the periosteum of the tooth and alveolar border of the palatine aspect. It is only two weeks since I saw that case, and I could not explain the reason for a tooth being drawn up except by union of periosteum and socket of that tooth and alveolar border. There can be no explanation, because it is not held there by anchylosis, and if there had been no union it would have dropped out.

*Dr. Sudduth*—May I ask the gentleman if it had other articulations—had an opponent?

*Dr. Gerhart*—It had.

*Dr. Sudduth*—Was the tooth too long?

*Dr. Gerhart*—There was occlusion of the two teeth.

*Dr. Sudduth*—If the shortening cannot be accounted for by occlusion, it may be because of the cicatricial tissue which surrounds the root, as the speaker says that there was not bony anchylosis in this case. In regard to the question of revivification of tissue, many experiments have been made. Tissues live longer than might be supposed; but the length of time is counted by hours, and not by days or years. Many experiments have been made in skin-grafting. A friend of mine in Chicago sent clippings of skin from the surgical cases occurring in the hospital daily to a friend of his in Wisconsin, who grafted them on an indolent ulcer. Some of them were detached as long as thirty-six hours, and were transported through the mails, yet grew. They were kept moist, and every precaution taken to treat them antiseptically. I do not for one moment believe in the theory of revivification of the pericementum on implanted roots. It's foreign to all our ideas of life.

*Dr. Faught*—I am very much interested in this subject, and have been since it was first introduced. As much as I have been in dental literature, and from the same standpoint, I must confess I have not had confidence enough to perform this operation, and while I do not want to say anything against the theory that would deter others, I still want to say that the saying, there is no danger, should be taken with a good deal of consideration. I do not think we ought to run blindly into the operation, even with the antiseptic treatment. Dr. Barker was an enthusiast in this matter. We remember the success he had, but in late years he said: "It is a thing I want to take back; it is a thing I want you all to very careful of." We all know the history of the case. I would hardly like to see the subject passed without raising my voice, even if I cannot raise it from a practical standpoint.

*Dr. C. B. Kratzer*—I would like to ask Dr. Kirk whether, in this operation, he pays any attention to the pathological conditions while it is removed from the socket. Now all of us extract teeth; the pericementum is often partially denuded from the root, and I should like to know whether Dr. Kirk would implant such teeth, or whether he wants perfectly sound teeth, and if the latter I think it will destroy the progress of conservatism in dentistry.

*Dr. Kirk*—In reply to Dr. Kratzer, as to whether I use any care in selecting the tooth, I thought I had covered that point in the paper. I suppose I have had quarts of teeth sent from various places to know whether they were good. I stated in the paper that they should have their pericemental covering intact. I certainly take the greatest care that they shall be perfectly healthy. As to destroying the principle of conservatism, I do not think it has any influence upon the subject. If a man is going to be influenced by it, he has no right to practice dentistry. The same might be said of whiskey and high license. I am sure I have not kept sober on account of the decrease in number of saloons. I am glad Dr. Kratzer has made the point he has, and I hope discussion will be brought about. I am earnestly and honestly seeking any evidence of harm that has or can result from this operation. Now I say that the gentlemen who make the severest criticisms or questioning as to the dangers or advisability of the operation are those who have not seen the operation. I am reasoning only from my own results, and with regard to the special operation Dr. Faught has referred to of Professor Barker's terminating so disastrously, we have heard all kinds of versions as to its cause. I want to know from somebody who knows everything concerning that operation and the cause of his unfortunate result, because I have had the version of so many that I do not know what did take place. In regard to Prof. Barker reconsidering his views of transplantation, he was equally rabid in regard to nitrous oxide, and yet we all know its value.

*Dr. Smith*—I have been one of those who have been fearful of treading the path of replantation or transplantation. I do believe there should be great caution taken in this matter, but I do not consider when a man tries a new thing that he is foolish by any means ; but there may be fools who might undertake it, and, therefore, Mr. President, we should be very cautious in working upon a variety of diatheses. It has been spoken to-day that we must remember we are working upon living tissues with dead matter, and therefore we should be careful, and Dr. Barker has left us in his memoirs something to think about and to ponder over. I remember something about the case, but I would not like to go into details ; but to cover the whole matter, it was a matter of diathesis. The trouble Dr. Barker had in his hands turned out to be tetanus, and, of course, was likely to lose the patient's life. It occasioned a great deal of discussion at the time, and especially by Dr. Barker, who had cautioned the young and old of the profession to be careful in this matter—to know whom they were going to operate upon, and I would caution Dr. Younger, if he is present here to-day, and the doctor who wrote the paper, to be very careful whom they select, for they are going to drill into a jawbone. The work may be done excellently ; but there are contingencies in the matter, and he must take precaution. I know every professor, or any man of experience here, will bear me out in the saying, to be careful in these matters, for if there should a death occur, a great loss of patients would result. It not only reflects upon the man but upon the profession, and we do not want to be the laughing stock of the medical profession. Now, in the growth of tissue, I believe that as long as there is a living cell there we will have growth, but when the cells are dead there will be no more growth. If you stick a branch of a tree in the ground—I stuck one in the ground a long time ago, and did not think it would grow—but it has taken root. There were no roots, but there were cells there. But where we have a tooth root that has been out six months or nine years it is dead matter, and never can grow; and as my friend awhile ago rightly said, it is not a matter of growth, but of ankylosis.

*Dr. James Truman*—I do not want to say much upon this subject, for I know little about it; but the matter brought to mind of Dr. Barker, a colleague of mine, occurred ten years ago, and it has become dental history. I do not regard the two cases at all as the same. Dr. Barker, as you know, removed four or five teeth from a lady under supposition that there were growths at the roots—nodular deposits. He replaced these, and the last tooth he replaced caused the death of the patient. What were the conditions of that period? Antiseptics were scarcely known. He probably used nothing in the way of an antiseptic or germicide upon the necks of those teeth. If he had known it sufficiently at that period, the case would not have resulted as it did. Then, again, there was more or less irritation. The periosteum was in place, and the pericementum was also in place; and placing a tooth back under those conditions was different from the plan of Dr. Younger, as illustrated by Dr. Kirk this afternoon. I have seen a number of cases, and I cannot see the great danger that is attributed to it. The cases are not synonymous at all. The only danger I do see in the bicuspid teeth is entering into the antrum. It struck me that, in careless hands, this might occur; and I should think it would cause great irritation during the process of healing. I believe that Dr. Sudduth has covered the whole ground in regard to the fastening of the teeth, and it occurs to me why use natural teeth at all? Why not use porcelain teeth made with depressions or screw-like forms? and then we would get rid of all danger of transmitting disease from one patient to another.

*Dr. Guilford*—There are one or two things about this operation that have not been brought out. In regard to this operation, we did not know what had induced Dr. Younger to begin; and he being a Californian and we here, most of us said, "There is another fool;" because we thought that we knew all about it, and that he did not. When he came East, there were very few places where he got to operate upon patients. Practitioners would not go, and said it was unphysiological. But when Dr. Younger inserted a tooth in the process, and it grew fast, there was evidence that something had taken place to make it unite to this tissue. Then came the question, How can this be done? Dr. Younger said that the tooth should be covered with pericementum. As Dr. Smith said when the tooth is dead there can be no life, Dr. Younger could not tell how the tooth was held, and thought it had a living attachment. How could it be when the pericemental membrane was dead? He did not know; but was willing to let them examine the case and see for themselves, and explain why this took place. Dr. Atkinson took a small bistoury and passed it up; and when he withdrew it there was blood, and said there was a living attachment. He was both wrong and right. The question of the whole subject is, What sort of union takes place? Dr. Kirk has given the best hypothesis in regard to this, and, to make it brief, it is about this: that while the pericemental membrane cannot be revivified, it can act as a leader to the tissue and into the opening into the cementum; and while it is held there, it is not because it fits the root, but has little processes running into the cementum that hold it there. If you use porcelain teeth, the living tissue will not get into it, and consequently will not bring about union. There perhaps may not be a living for that socket; but there is a healthy growth there which takes hold of this dead pericementum, which acts much like a sponge; but with the smooth surfaces of the porcelain tooth the same results would not be produced.

*Dr. Kingsbury*—I find that Dr. Truman has anticipated a line of thought, and remarks I had in mind, particulary in reference to the case referred to, that of Dr. Barker. I was conversant with the case. Dr. Barker and myself were very well acquainted, and one day he called at my office, stated the case to me, and desired me to come around to the Pennsylvania College, and hear his statement of the case, and that a number of the members of the profession were invited to be present. The fact is, Dr. Barker had become apprehensive of some unfortunate and, perhaps, fatal results. He made a statement of the case and, as far as possible, received the indorsement of the medical profession, and he desired, as was natural, to receive the endorsment of the dental profession. The operation was a very unfortunate one; but there was no simularity between that operation and the one now practiced by Drs. Younger and Kirk. In Dr. Barker's case five or six teeth were extracted, one after the other, as a remedy for neuralgia. After all efforts failed to give her relief, he concluded to extract the anterior superior teeth and excise the point. He did so and, upon inquiry, he stated to me, he did not file off or smooth in any way the ends of those roots after they were excised, but left them with the sharp points and cutting edge; and I am quite sure that the roots inserted in the sockets becoming such a severe excitant to the general nervous system produced the tetanic effect which resulted in her death. It was hushed over as much as possible. The sympathy of the profession was with Dr. Barker. He was experimental in his way, and was prone to efforts for advancement in science, and this particular case was a fatal advance; but I think there is very little danger from the operation of Dr. Younger. When I first heard of the operation of Dr. Younger, my mind reverted to other operations somewhat similar in their character. In the first year of my practice in extracting a molar, I extracted the wisdom tooth also, the roots of the second molar were united with the roots of the second molar in such a way that the latter was forced out with the second molar, and perhaps a little inexperience had something to do with it. I replanted it, and it became very firm, and the last I heard of the tooth, it was a useful tooth in the mouth. The operation of transplantation of teeth is more than one hundred years old. It was very popular amoung French dentists at one time, and has been done in this country, and there have been many, more or less, disastrous consequences by the inoculation of disease from one person to another. I have been very much interested in it, and regard it as a phenomenal operation, a marked indication of advancement. I would not attempt to explain how a tooth that has been deprived of its vitality, and inserted into a living tissue, will become so firm and hard, as we find is these cases. I had an opportunity to examine a number of cases, several performed by Dr. Kirk, and I have been astonished at the firmness of the teeth in the maxilla. I did not think the pericemental membrane is essential to the success of this operation, as seems to be inferred from the remarks of Prof. Guilford, but believe that even if the tooth was deprived of the covering, it should be as successful. I do not see why it should not, and I would like to ask if it is essential. If you scrape off the pericemental tissue, would not the chances be as favorable for the operation?

*Dr. Kirk*—I think not; but I simply speak from a clinical standpoint, and not from any theory which I have. Dr. Younger has had several teeth implanted in his own mouth, and one that I saw extracted about a year ago was much absorbed upon one side, and was altogether in an unfavorable condition; so much so that I thought there was no doubt of its ultimate failure had it been left for a time. Dr. Younger told me that the tooth he used in that case had a defective membrane. A microscopical examination developed the fact that while there was good union on one side of the root, upon the other absorption had made deep inroads upon the cementum. While I do not have any faith in the theory that the pericemental membrane is in any sense revitalized, I am rather inclined to the idea that a tooth with its membrane adherent and perfect is a source of less irritation to the tissues when introduced, and the process of healing is thereby more favored than it would be if the membrane were removed or imperfect.

*Dr. Kingsbury*—That is quite an important point to be borne in mind, but I think still it must be conceded that there is no more vitality in the pericemental membrane than there would be in the cementum itself. I cannot think there is any degree of vitality remaining in a tooth after it has been absent from the mouth for so long a time, and in the line of experiment I think it would be well to try scraping off the membrane of the root, and insert it in the regular way by Dr. Younger's method, and see if the operation would not be as successful as with it.. I believe it would be, and if it would not be I could not favor the opinion of Dr. Truman that porcelain would work at all. If the membrane is necessary, then porcelain could not be used. I have not felt like going into this line of practice myself, and the chief reason is that I have not as much confidence in the value and permanence of the operation as many seem to have.

*Dr. Guilford*—I would like to remind Dr. Kingsbury that sponge has no life in it, and yet it fills an important part in the production of tissue.

*Dr. Litch*—I did not have the pleasure of hearing Dr. Kirk's paper, but it seems to me that there are certain states of the system that would make the operation a dangerous one. I would select very carefully my patient, being careful to avoid scrofulous conditions, etc. I am not fully convinced of the permanency of the operation. We must remember that the tissue holding it in place is cicatricial tissue, and is liable to accidents, and will sometimes melt away. Of course you must take those points into consideration in determining the operation, and the patient should be notified of the possible risk and failure. There has also been mentioned the danger of entering the autrum in the case of the bicuspid teeth. It would result in inflammation of the membrane of the autrum, and might produce necrosis. The operation of Dr. Barker's was performed under different conditions, and, as Dr. Kingsbury has remarked, the end of the root cut off, and not filled, I think that that condition largely accounted for the result.

### MORNING SESSION, JUNE 7TH, 1888.

### PAPER READ BY DR. W. E. MAGILL, ON "IRREGULARITIES AND THEIR CORRECTION."

### DISCUSSION.

*Dr. Guilford*—The only thing we can do with a subject of this kind is to take up one or two points the essayist has touched upon. One of these points is in regard to the extraction of teeth either as a preventive or corrective method. It is one of those things which requires the highest order of cultivated judgment, and also experience, because if it is done judiciously and wisely, it is one of the best things that can be done; injudiciously and unwisely, one of the most harmful. When people ask whether I extract one or the other, I say it all depends upon the surroundings. Very often we can postpone the extraction of a tooth until we can see what the results are, and then, if necessary, we can extract it. Individuals are sometimes in doubt as to whether they should enlarge the arch or extract the tooth. It is a hard question to decide, and hard to know what to do after the arch is expanded. There is another point which wants to be carefully looked at, and one I teach young men, and that is the inherent force of nature, a tooth out of position comes into position if the opportunity is offered. It only wants room or removal of the obstacle in its way. There are no teeth that possess this power as the cuspid teeth. If there is no room for them in the mouth, remove the bicuspid, and the cuspid comes into its place. It is the inherent force of nature. Some years ago, I had a patient, a young girl, who had a cuspid tooth outside the arch out of line. The second bicuspid was very good, and the molar somewhat bad. I said I would extract the first molar, and then bring back the bicuspid, and draw the cuspid into place. I put an appliance on the second bicuspid, and I was told the appliance hurt so much—ligatures, etc.—that the father cut it off, and decided to abandon the case. About two years after that she came back to have her teeth filled. I saw a beautiful arch. I said, Miss A., you have been to a dentist, and had your teeth regulated. She answered, no. I asked her, have you had nothing done to bring the teeth into place? She said, no. That cuspid tooth had forced the other tooth back, and come into place. I make this point to show the inherent force of nature, even against certain obstacles. There often is difficulty in the cuspid teeth where they do not erupt into line. If you can preserve a space for them, they will come through of their own accord. If we give nature a chance, she will do much for us, and very thoroughly.

In regard to interrupted and continuous pressure, Dr. Magill prefers the interrupted. I have studied this very much, and I have had good results in both ways. I have had good results quicker with the continuous. I think experience shows that the action of the metal spring, or anything else continuous in its character, will move the tooth all right. I find that in continuous pressure, leaving out soreness, there are no bad effects.

*Dr. Kingsbury*—Where the inferior incisors come anterior to the superior incisors, the sooner that form of irregularity is remedied the better. From the time of eruption of most teeth—say up to the period of ten years—from that time up from five to six years there is an enlargement of both jaws that many do not take into account in considering the question. While there may be a crowded condition of the teeth at ten or twelve years, five or six years afterward this irregularity may disappear. I regard it as a radical error for a dentist to extract a cuspid tooth except in marked cases of irregularity. If the first bicuspid is extracted, there is usually no difficulty, with the enlarged growth of the jaw during the succeeding year, in bringing the cuspid down into its position. In a like case it is good to extract a second bicuspid to meet a difficulty of this kind, rather than the first one; because the second is more liable to caries than the first. In all cases where the second is extracted, the first will recede, and the cuspid come into position.

*Dr. Chupein*—I was about to make the same remarks that Dr. Kingsbury made when he arose before me. I think one of the most important cases that call for the interference of the dentist is when the superior teeth close inside of the lower teeth. Such a deformity will not be rectified by extraction of any teeth, and will only be met by the interference of the dentist.

